# Supporting the Management of Gestational Diabetes Mellitus With Comprehensive Self-Tracking: Mixed Methods Study of Wearable Sensors

**DOI:** 10.2196/43979

**Published:** 2023-10-31

**Authors:** Mikko Kytö, Saila Koivusalo, Heli Tuomonen, Lisbeth Strömberg, Antti Ruonala, Pekka Marttinen, Seppo Heinonen, Giulio Jacucci

**Affiliations:** 1 Helsinki University Hospital IT Management Helsinki University Hospital Helsinki Finland; 2 Department of Computer Science University of Helsinki Helsinki Finland; 3 Department of Gynecology and Obstetrics Turku University Hospital Turku Finland; 4 Department of Gynecology and Obstetrics University of Turku Turku Finland; 5 Department of Gynecology and Obstetrics Helsinki University Hospital Helsinki Finland; 6 Department of Gynecology and Obstetrics University of Helsinki Helsinki Finland; 7 Department of Computer Science Aalto University Espoo Finland

**Keywords:** gestational diabetes, self-management, self-tracking, wearable sensor, mobile application, self-discovery, behavior change, user experience

## Abstract

**Background:**

Gestational diabetes mellitus (GDM) is an increasing health risk for pregnant women as well as their children. Telehealth interventions targeted at the management of GDM have been shown to be effective, but they still require health care professionals for providing guidance and feedback. Feedback from wearable sensors has been suggested to support the self-management of GDM, but it is unknown how self-tracking should be designed in clinical care.

**Objective:**

This study aimed to investigate how to support the self-management of GDM with self-tracking of continuous blood glucose and lifestyle factors without help from health care personnel. We examined comprehensive self-tracking from self-discovery (ie, learning associations between glucose levels and lifestyle) and user experience perspectives.

**Methods:**

We conducted a mixed methods study where women with GDM (N=10) used a continuous glucose monitor (CGM; Medtronic Guardian) and 3 physical activity sensors: activity bracelet (Garmin Vivosmart 3), hip-worn sensor (UKK Exsed), and electrocardiography sensor (Firstbeat 2) for a week. We collected data from the sensors, and after use, participants took part in semistructured interviews about the wearable sensors. Acceptability of the wearable sensors was evaluated with the Unified Theory of Acceptance and Use of Technology (UTAUT) questionnaire. Moreover, maternal nutrition data were collected with a 3-day food diary, and self-reported physical activity data were collected with a logbook.

**Results:**

We found that the CGM was the most useful sensor for the self-discovery process, especially when learning associations between glucose and nutrition intake. We identified new challenges for using data from the CGM and physical activity sensors in supporting self-discovery in GDM. These challenges included (1) dispersion of glucose and physical activity data in separate applications, (2) absence of important trackable features like amount of light physical activity and physical activities other than walking, (3) discrepancy in the data between different wearable physical activity sensors and between CGMs and capillary glucose meters, and (4) discrepancy in perceived and measured quantification of physical activity. We found the body placement of sensors to be a key factor in measurement quality and preference, and ultimately a challenge for collecting data. For example, a wrist-worn sensor was used for longer compared with a hip-worn sensor. In general, there was a high acceptance for wearable sensors.

**Conclusions:**

A mobile app that combines glucose, nutrition, and physical activity data in a single view is needed to support self-discovery. The design should support tracking features that are important for women with GDM (such as light physical activity), and data for each feature should originate from a single sensor to avoid discrepancy and redundancy. Future work with a larger sample should involve evaluation of the effects of such a mobile app on clinical outcomes.

**Trial Registration:**

Clinicaltrials.gov NCT03941652; https://clinicaltrials.gov/study/NCT03941652

## Introduction

### Background

Gestational diabetes mellitus (GDM), defined as hyperglycemia first recognized during pregnancy, is an increasing global challenge currently affecting approximately 8%-23% of pregnancies depending on the continent [1]. GDM has considerable health effects as it increases the risk for short- and long-term health disadvantages among both the mother and child [2]. Although GDM is a temporary condition that lasts until the birth of the child, it increases the later risk of type 2 diabetes for mothers by over 7 times [3]. Healthy lifestyle choices help in GDM management, with nutrition being the primary factor affecting glucose levels [4], and physical activity [5-9], stress [10], and sleep [11] also have impacts on glucose homeostasis. However, women with recently diagnosed GDM do not adequately know how their own lifestyle choices influence glucose levels [12,13], although they need to adapt to the new situation quickly [14]. Given that pregnancy usually lasts approximately 40 weeks and GDM is diagnosed after 12 to 28 weeks of pregnancy, any health intervention designed for managing GDM can be used for a limited time (for approximately 12-28 weeks). On the other hand, women with GDM show extra motivation for managing diabetes owing to the child [13,15], and pregnancy represents an exceptional opportunity for lifestyle changes [16].

A recent meta-analysis of eHealth interventions targeted to women with GDM showed that interventions providing weekly or more frequent feedback from health care professionals to women with GDM have the potential to improve glycemic control [17]. Typically, in these interventions, women with GDM can communicate with the study interventionists remotely [18,19]. For example, a recent study by Miremberg et al [18] revealed a statistically significant improvement in glycemic control among women with GDM when systematic feedback was provided by study personnel (every evening the participants received individualized feedback via email from the clinical team regarding their daily glycemic control). However, mobile health (mHealth) interventions without such substantial input from health care professionals are limited and have not been shown to be effective [20,21]. We expect that the effectiveness of mHealth interventions can be increased with comprehensive self-tracking through wearable sensors by providing more insights for women with GDM into learning associations between lifestyle and glucose levels [22,23], a process known as self-discovery (eg, [24]).

To establish knowledge on how self-tracking with wearable sensors (including glucose levels and lifestyle) should be designed to support self-management in GDM, we explored the usage of continuous glucose monitors (CGMs) and 3 types of wearable sensors for measuring physical activity. The overall aim was to examine how wearable sensors can support self-discovery and behavior change, and how women with GDM experience them.

### Wearable Sensors for Supporting Self-Discovery for Women With GDM

Wearable sensors (eg, fitness trackers) have been included in investigations on the management of noncommunicable diseases, such as diabetes, migraine, and multiple sclerosis [25-32]. Moreover, in pregnancy, a recent review showed that wearable sensors have the potential to support physical activity among pregnant women, decrease gestational weight gain, predict neonatal outcomes, and support monitoring of fetal heart rate and movements [33]. However, there are no studies where the focus is on investigating how different wearable sensors (eg, in terms of body placement) and their data can support self-discovery. Traditionally, studies on personal discovery in diabetes management have been based on the data that users enter into an app [34] or write in a paper-based journal [24].

The personal discovery of understanding medical conditions with self-tracking data has gained a lot of attention [24,25,27,29,35-37]. Personal discovery is an iterative and complex process consisting of multiple stages [24,26,35]. These stages include finding potential features that may affect the desired outcomes, forming hypotheses, and evaluating their impacts on outcomes [24,38]. In diabetes, successful self-management requires knowledge of how one’s activities and lifestyle (eg, nutrition, physical activity, sleep, and stress) affect glucose levels. To help people with diabetes in self-discovery, self-tracking with wearable sensors together with glucose monitoring may provide a useful tool. However, the role of self-tracking of activities and lifestyle together with glucose levels using wearable sensors in the self-discovery process is largely unknown. For example, while physical activity and sleep have been found to influence glucose levels [6,8,11] and a handful of wearable sensors for measuring physical activity and sleep are available for self-tracking, the applicability of wearable sensors in supporting the understanding of people with diabetes about how their own lifestyle choices affect glucose levels is largely unknown.

Women with GDM represent an interesting user group to study self-discovery, as they have not been managing their condition for long. The design of supporting the discovery phase becomes an especially important part of the management of GDM, as “coming to terms with GDM” and learning new strategies for self-regulation are important phases in GDM self-management [13,15]. Qualitative studies have reported feelings of failure, anxiety, loss of control, and powerlessness after receiving a GDM diagnosis [13,14]. However, women with GDM experience “a steep learning curve,” and they move from the initial shock of the diagnosis to acceptance and active management of their condition [39].

For women with GDM, it is typical to find associations between nutrition and blood glucose by trying out different foods and measuring glucose afterward [13,39,40]. The behavior where patients try to establish hypotheses between daily activities and changes in disease-specific outcomes has been identified as a stage-based discovery process [24,35,38].

The framework from Mamykina et al [24] has been formulated to explain the discovery process between daily activities and changes in blood glucose levels. According to the framework [24], self-discovery consists of the following 4 stages: (1) feature selection (individuals identify activities that they believe have an impact on outcomes, eg, blood glucose in the context of diabetes); (2) hypothesis formulation (individuals formulate suspected associations with activities and outcomes); (3) hypothesis evaluation (individuals observe new information about their condition and evaluate how it fits to already collected data); and (4) goal specification (individuals formulate future goals based on identified relationships between features and outcomes).

Multiple studies have emphasized the importance of automatic data collection in diabetes apps [22,41], although this is rarely found in apps used in diabetes research [22,41]. Current standards emphasize the necessity of self-tracking glucose levels in diabetes management [5], and measurement of blood glucose levels has been found to be the most important feature of a GDM app [42]. However, the requirement of manually entering blood glucose values has decreased significantly for collecting glucose data [42,43]. Glucose measurements can be performed automatically and more frequently with CGMs. CGMs have been found to be acceptable among women with GDM [44-47]. However, recent research suggests that a CGM alone does not improve glycemic control [45,48] or decrease macrosomia [47]. One reason is that the cause and effect between lifestyle choices and glucose levels are not clear for women with GDM after receiving a diagnosis [13-15,39,40].

While self-discovery frameworks have been critiqued for expecting too rational and coherent behavior from people using self-tracking [25] (users are not scientists [49]), the trial and error aspect (hypothesis formulation and evaluation) has been identified as typical behavior among women with GDM [13,39,40]. Moreover, the framework by Mamykina [24] also considers the iterative nature of self-discovery, which is important in the context of GDM, as the development of pregnancy has an impact on glucose control [50]. Objectively and automatically measured and constantly available data obtained through wearable sensors can be expected to support self-discovery [26,27].

### User Experience With Wearable Sensors for Women With GDM

Self-tracking is often mentioned as an effective behavior change technique [51], for example, shown as increased physical activity among people with type 2 diabetes [52]. Thus, we investigated the possibilities and challenges of self-tracking with wearable sensors beyond self-discovery. Wearable sensors have the potential to facilitate the management of GDM, as there is proof that lifestyle interventions using wearable sensors can be effective among pregnant women. For example, Chan and Chen [53] reported in their review that interventions with wearable devices for increasing physical activity were more effective than those without wearable devices among pregnant women.

Physical activity is one of the cornerstones in the management of GDM [5,7], but the automatic collection of physical activity data has gained minimal attention in GDM apps [22]. This was emphasized in a study by Skar et al [42] who asked women with GDM to manually enter their physical activity data into an app, but no participant entered the data, preventing the collection of any physical activity data. This is understandable as pregnant women often have limited energy for monitoring their own behavior, since they already have a lot to do and deal with [40,54]. Rigla et al [55] enabled tracking of physical activity for women with GDM by recording the activity with an accelerometer in a mobile phone. However, recording required manual start and stop by pressing buttons in a mobile app, and participants recorded their physical activity only approximately once a week on average. Even engagement with automatic self-tracking has been shown to decrease among people with type 2 diabetes and type 1 diabetes [56]. For example, Böhm et al [56] reported that the number of active users of CGMs dropped by over 20% after 20 weeks, and similarly, active users of automatic physical activity tracking dropped by over 30% after 20 weeks.

The other issue to consider in addition to the automaticity of tracking is what types of physical activities are possible to track. Carolan et al [15] noted that although walking is commonly advised for women with GDM by diabetes educators and midwives, it can be painful for many. However, automatic self-tracking beyond steps is more challenging. Årsand et al [30] found that the largest problem for people with type 2 diabetes to track their physical activity was that wearable sensors did not support the measurement of other activities, such as cycling and swimming, which are common physical activities among pregnant women [57]. More recent studies implied that wearable sensors have still rather low validity in tracking physical activities beyond walking and running, such as bicycling and resistance training [58].

Studies investigating the practical challenges of wearable sensors for self-tracking among women with GDM are largely lacking. As described above, only few studies have enabled self-tracking of physical activity among women with GDM [42,55], and in the case of self-tracking of other lifestyle factors (eg, sleep and stress) with wearable sensors, no studies have investigated self-discovery among women with GDM. In the context of pregnancy, automatic self-tracking of lifestyle (eg, nutrition, physical activity, and symptoms) has been argued to help in countering pregnancy-related health risks [59,60]. However, some women perceive pregnancy medicalization and state that they lack control over their own bodies even without multiple wearable sensors [13,54]. The use of sensors can further increase the feeling of losing a normal pregnancy [13]. Moreover, it is unclear how the sensors fit pregnant women whose physical and mental conditions are different from those of the general population. Pregnancy causes several lifestyle changes (eg, diet limitations), physical changes (eg, difficulty moving, contractions of the uterus, and increased waist size and heart rate), sleeping disorders, and tiredness. The effect of differences in these conditions on self-tracking with wearable sensors should be investigated.

## Methods

### Research Design

We conducted a mixed methods study where women with GDM (N=10) used a variety of wearable sensors and their mobile apps for a week. Our primary aim was to examine how wearable sensors can support the self-management of GDM. We studied this with 2 research questions (RQs) as shown in Textbox 1. We investigated how self-tracking with wearable sensors can support or inhibit self-discovery (RQ1) and how women with GDM experience wearable sensors (RQ2). The study was performed in Finland.

**Research questions.**

- Research Question 1: How self-tracking with wearable sensors (not only continuous glucose monitors) can support or inhibit the self-discovery of women with gestational diabetes mellitus (GDM)? We investigated the role of wearable sensors at each stage of the self-discovery process (feature selection, hypothesis formulation, hypothesis evaluation, and goal setting), as described in the section Wearable Sensors for Supporting Self-discovery for Women With GDM.
- Research Question 2: How do women with GDM experience wearable sensors? Although wearable sensors have been investigated with pregnant women and people with type 1 or type 2 diabetes, the knowledge of how women with GDM perceive wearable sensors is less known, as described in the section User Experience With Wearable Sensors for Women With GDM.

### Ethical Considerations

The study was performed in compliance with the Declaration of Helsinki and was approved by the Ethics Committees of Helsinki Central Hospital (September 14, 2006; Dnro 300/E9/06). The study was registered at Clinicaltrials.gov (NCT03941652).

### Sensors

#### Continuous Glucose

**Figure 1:**
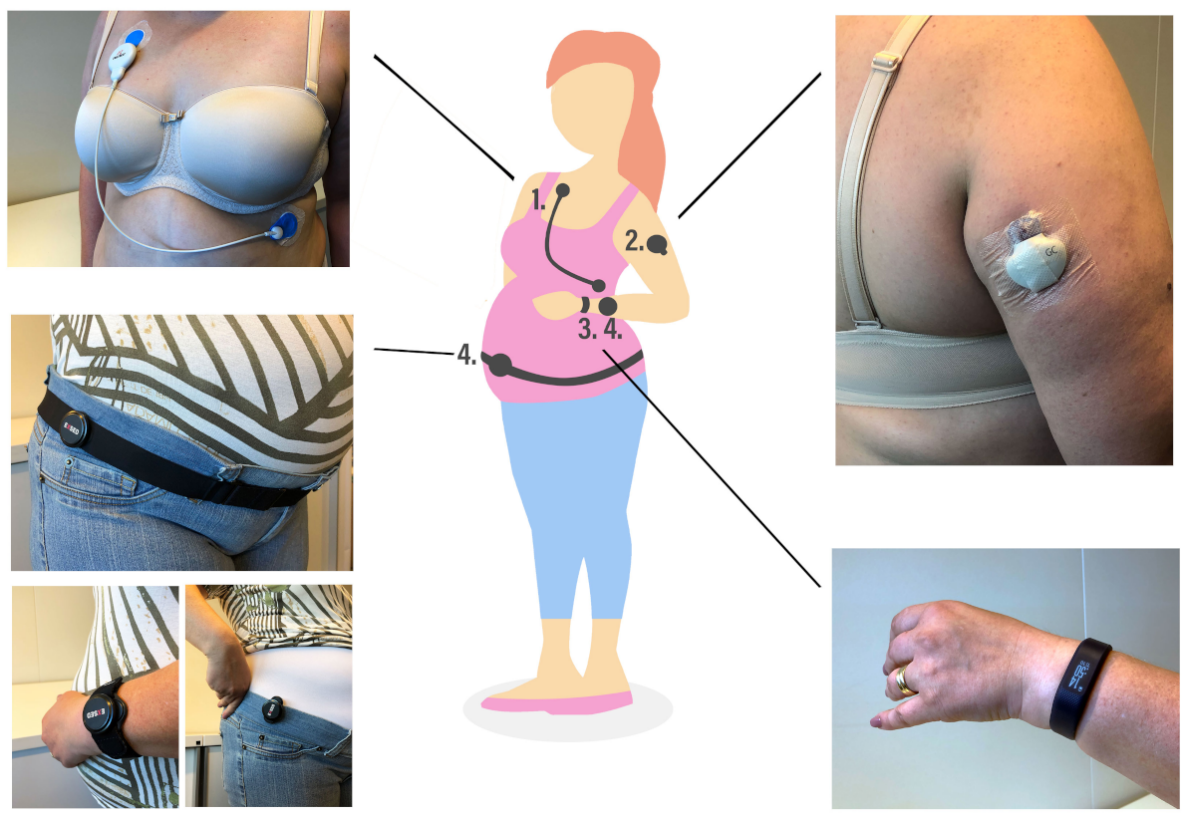
The wearable sensors used in the study: (1) Firstbeat, (2) Medtronic Guardian Connect, (3) Vivosmart 3, and (4) Exsed.

Medtronic Guardian Connect CGM with an Enlite sensor (Medtronic; Figure 1) can continuously measure tissue glucose. A flexible filament is inserted just under the skin to measure glucose levels in interstitial fluid every 5 minutes. Values are sent to the Medtronic Guardian app via Bluetooth. If a Bluetooth connection is not possible, the CGM system transmitter collects the data for several days. Medtronic requires calibration of the sensor through fingertip blood glucose measurements 2 times a day. The overall mean absolute relative difference has been reported to be 13.6% [61].

The Medtronic CGM was attached to the skin by a study nurse. This was because participants wished to wear the CGM on the arm and they could not attach the CGM to the skin using only one hand. Currently, CGMs do not allow tracking of lifestyle data, and additional sensors are needed to support tracking beyond glucose.

#### Physical Activity

We chose to use multiple physical activity sensors to study which sensor or combination of sensors should be used in terms of wearing comfort and provided data. Details are provided in Figure 1 and Table 1. Exsed (UKK-Institute) was worn on the hip and provided data about standing and sitting. The data analysis was based on validated MAD-APE algorithms [62,63]. These analyses have been employed in population-based studies of Finnish adults [64,65]. Vivosmart 3 (Garmin) was worn on the wrist and provided data about intensity minutes. Vivosmart 3 has been shown to measure steps well at slow walking speeds (mean absolute percentage error was 1.0%) [66], which is important as walking speed is affected by pregnancy [67].

Physical activity sensors also varied in terms of how visible they were to others nearby. Exsed could be worn in a discreet manner so that others would not see it, whereas Vivosmart 3 was worn on the wrist and was more conspicuous. This *physicality* has been shown to be a prominent issue for wearable sensors [49].

The heart rate variability (HRV) sensor Firstbeat Bodyguard 2 (Firstbeat Technologies) was added to explore the validity of physical activity and sleep data recorded with physical activity sensors. The device is able to continuously measure beat-to-beat HRV with an error of <3 ms and a detection rate of >99.9% as compared with clinical-grade electrocardiography [68].

Due to incompatibility issues between different operating systems and sensors, the participants were given an iPod touch with the sensor apps preinstalled. The participants were able to use their own mobile phones with Vivosmart 3, as we found no incompatibility issues in the Garmin Connect app prior to the study.

**Table 1:** Wearable sensors worn by the participants (participants wore all sensors simultaneously).

Sensor name	Type	Data provided	Wearability	Components	User interface	Waterproof	Worn by each participant
Medtronic Guardian Connect CGM^a^ with an Enlite sensor	CGM	Interstitial fluid glucose value every 5 minutes	Typically worn on the area of the abdomen, which is at least 5 cm from the navel, but participants wished for attachment to the upper arm.	Enlite sensor: flexible filament measures glucose levels in interstitial fluid; Guardian Connect transmitter: Bluetooth	None. Data access through a mobile app (Medtronic Guardian Connect). The app enables viewing the time series of glucose values, and the viewing range can be changed from 1 hour to 1 day. Users insert the calibration values twice a day, and it is possible to add carbohydrates and physical activities to the timeline.	Yes, up to 2.5 meters for up to 30 minutes	Mean=94% of the time (23 h and 3 min/day)
Garmin Vivosmart 3	Activity tracker	Steps, intensity minutes, stairs climbed, heart rate, sleep duration, sleep quality, stress, and calorie consumption	Worn on the wrist with an adjustable plastic strap.	Bluetooth Smart, ANT+, 3D accelerometer, optical heart rate sensor (green LED), barometric altimeter, and ambient light sensor	Touch screen, and data access through a mobile app (Garmin Connect). The app enables viewing of many kinds of information about the recorded data, and the time span of the graphs can be varied between 1 day and 1 year.	Yes, up to 50 minutes	Mean=93% of the time (22 h and 30 min/day)
Exsed	Activity tracker	Duration of physical activity, sedentary behavior, sleep sensor, sitting, standing, breaks in sitting, steps, sleep duration, and sleep quality	Worn on a belt around the hip or on a clip attached to trousers, and worn on the wrist during nighttime.	Bluetooth, 3D accelerometer, and gyroscope	None. Data access through a mobile app (Exsed2). The app visualizes the recorded data on a daily graph and a weekly graph.	Yes, up to 30 meters	Mean=83% of the time (19 h and 55 min/day)
Firstbeat Bodyguard 2	HRV^b^ sensor	Stress, recovery, duration of physical activity with intensities, HRV, heart rate, excess postexercise oxygen consumption, respiration rate, and others	The device is attached to the chest with 2 disposable clinical-grade electrocardiography electrodes.	3D accelerometer and beat-to-beat heart rate	None. Data are provided in a PDF after the measurement period.	No	Mean=93% of the time (22 h and 30 min/day)

^a^CGM: continuous glucose monitor.

^b^HRV: heart rate variability.

### Recruitment and Data Collection

Our goal was to recruit 10 women with GDM from maternity and antenatal clinics in the Helsinki Metropolitan Area (Finland). The goal for the number of participants is similar to that in multiple qualitative studies on women with GDM [12]. The clinic nurse asked women with GDM at least at 24 gestational weeks about their interest in participation. If interested, the study nurse contacted the mother with more information about the study and confirmed eligibility. The exclusion criteria were type 1 or type 2 diabetes, use of medication that can influence glucose metabolism (eg, oral corticosteroids, metformin, and insulin), diagnosis of GDM in previous pregnancies, current substance abuse, presence of a severe psychiatric disorder, significant difficulty in cooperating (eg, inadequate Finnish language skills), and significant physical disabilities that would prevent the use of a smartphone or moving without aid. Data were collected using the following procedure. After obtaining informed consent, we collected background information (eg, age, pregnancy weeks, and familiarity with mobile apps) through a questionnaire.

Participants were asked to wear wearable sensors (see the section Sensors) for 6 days and nights, after which they were interviewed in their native language (Finnish). The length of the usage period was decided based on the battery life of the transmitter of the CGM, which was 6 days. To compare data from wearable sensors with their perception of physical activity and sleep, participants filled out a logbook for physical activity and sleep (duration in hours) for 6 days. For physical activity, participants were asked to write down the type of activity, duration, and intensity (light, moderate, or vigorous). The perceived intensity levels were defined according to descriptions by Norton et al [69]. Moreover, Firstbeat used the same intensity categorization as provided in [69]: 20%-40% of maximal oxygen consumption (VO_2_ max) is considered light physical activity, 40%-60% of VO_2_ max is considered moderate physical activity, and over 60% of VO_2_ max is considered vigorous physical activity. Vivosmart 3 shows the intensity of physical activity as intensity minutes, which is gathered when physical activity at a moderate level is performed for at least 10 consecutive minutes. Physical activity at a vigorous level doubles the gathered intensity minutes. Explicit thresholds for moderate and vigorous activities are not provided in the documentation. Exsed did not provide data regarding the intensity of physical activity.

One of the most prominent features is tracking and managing diet, as this is the primary factor that affects glucose levels. However, wearable eating detection systems are not able to detect the macros of food [70,71]. As such, wearable sensors were not used to measure diet, and participants kept a diet logbook for 3 days during the study period. We chose to gather diet data for 3 days, because keeping a food diary is laborious and it has been shown that diet data for 3 days provide valid results [72].

Before starting the measurement period, participants were met by an experimenter and a study nurse. In the meeting, participants provided written consent, filled in a background questionnaire, and were instructed on how to use the sensors. They were given contact information in case they faced problems in using the sensors. Finally, at the end of the meeting, participants filled in a technology acceptance questionnaire based on the Unified Theory of Acceptance and Use of Technology (UTAUT) [73], which has been widely used for evaluating the acceptance of technology in diabetes management [74]. After the usage period, participants filled out the same UTAUT questionnaire and took part in a semistructured interview, which was audio-recorded. At first, we asked questions concerning all the sensors, such as how they impacted the users’ daily lives. After this, we asked questions concerning each sensor, such as what the users were able to discover from the data, how the data impacted their daily behavior, what data they valued, and what challenges they had with each sensor. See Textbox 2 for the main interview questions. Interviews were conducted in quiet places that were easiest for the participants to arrive at and were conducted in their mother tongue. Interviews lasted approximately 1 hour on average. After a 15-minute break, participants continued with an interview about a prototype GDM application (results are reported elsewhere [23]).

**Main interview questions regarding the wearable sensors.**

**Main questions about self-discovery**

- Have you made deductions based on the data from the sensors and their apps? If yes, what kind of?
- Has the usage of the sensors influenced your behavior? If yes, how?
- Do you think that the <sensor name> would help you to manage blood glucose? Please justify.
- Has the information from the sensors or their apps been confusing or unclear? If yes, what?
- Did you feel that the information from the sensors described your behavior truthfully?

**Main questions about the user experience**

- What factors influenced wearing the sensors?
- Have the sensors or their apps caused you any discomfort or inconvenience? If yes, which sensors or apps and how?
- Think about your experience with the sensors and their apps. How would you improve them?

### Analysis

Interviews were transcribed, and 2 researchers familiarized themselves with the interviews by reading the transcripts. The analysis was performed according to the framework method, which is a recommended approach for multidisciplinary health research [75]. We used self-tracking of blood glucose, diet, physical activity, sleep, and stress as initial codes. Coding was implemented with Atlas.ti by employing emergent theme analysis of the data collected [76], resulting in 66 codes altogether. These codes were combined into larger categories, which are presented and discussed in relation to the main themes of the study (ie, self-discovery and experiences with wearable sensors).

Quotes provided in the results were translated into English intelligent verbatim, a process whereby filler words such as “er” are removed during translation. Log files from the sensors were used to determine how much the participants wore them, how data from the sensors correlated with self-reported data, and whether there were differences in data between the sensors. The statistical significance of differences in data between sensors was computed with the Friedman test, and correlations between automatically measured and self-reported data were calculated with Spearman or Pearson correlations, depending on the test for normality (Shapiro-Wilk). Finally, we triangulated among these data sources (interviews, data from the sensors, and logbooks) to understand how self-tracking with wearable sensors should be designed to support self-discovery.

## Results

### Participants

Ten women with GDM (Table 2) were recruited. We had a variety of participants in terms of age (minimum 24 years, maximum 40 years). Participants were familiar with mobile apps and measuring glucose, but they had less experience with using wearable physical activity sensors, as depicted in Table 2. The same participants participated in another study after this study [23]. The mean age of the participants was 33.6 years, which is similar to that of women with GDM in Finland (mean 32.5, SD 5.3 years) and in the Helsinki area (mean 33.1, SD 5.1 years) [77]. The mean BMI of the participants was 25.7 kg/m^2^, which is in the range of the mean BMI of women with GDM in the Helsinki area (mean 27.1, SD 6.0 kg/m^2^) and in Finland (mean 28.5, SD 6.3 kg/m^2^) [77].

**Table 2:** Participant demographics and their experience with mobile apps and sensors.

ID	Age (years)	Weeks of gestation	BMI before pregnancy (kg/m^2^)	How many minutes per day do you exercise at a moderate level?	I am used to using various mobile apps^a^	I am used to using physical activity sensors (such as Fitbit, Vivosmar, and Polar)^a^	I am familiar with measuring blood glucose^a^
1	36	35.0	22.2	150	4	3	5
2	32	33.3	30.1	4	4	2	4
3	40	31.2	23.1	120	4	2	4
4	24	33.7	29.8	240	5	2	5
5	31	35.6	26.0	3	4	2	4
6	31	30.3	21.0	210	5	1	4
7	32	36.6	20.2	3	2	1	5
8	36	37.0	25.4	120	5	5	5
9	35	34.8	22.9	120	5	1	4
10	39	28.1	36.6	150	5	5	5
Mean	33.6	33.6	25.7	25.7	4.3	2.4	4.5

^a^For the statements, the Likert scale ranged from 1 (strongly disagree) to 5 (strongly agree).

### Factors Supporting Self-Discovery (RQ1)

#### Continuous Glucose Monitoring

While participants were familiar with measuring their glucose levels (Table 2), they learned new things owing to continuous monitoring.

I wish I had this [CGM] when I got the GDM diagnosis, so I would have got some knowledge of the glucose curve.

Participant #9

They learned new causalities between food and glucose levels.

I think it is better to have the data from 24 hours. Then you can see what happens in between. Nowadays, I eat nuts because I know that when I started eating nuts, my blood glucose started to be at a good level.

Participant #1

Improved glucose control was noted in Participant 2, who started monitoring glucose levels continuously and learned to adjust eating accordingly.

I had a couple of hypers [hyperglycemia], but I think with normal measurements those would not be noticed because they were irregular...especially the hypers in the morning...At first, I was like I don’t have any problem with them [glucose levels] but when you had that continuous measurement I figured out that it is not actually the case.

Participant #2

The CGM facilitated monitoring the variability of glucose, and among 7 of the 10 participants, the variability of glucose values decreased, which was calculated as a trend in the variability of glucose using LAGE (large amplitude of glucose excursions) [78]. The CGM not only supported self-discovery but also improved motivation to change the diet.

...you are able to see it [glucose] for the whole day...it motivates for changing the diet.

Participant #2

While participants had extra costs from wearing the CGM (see the section Wearing the CGM on the Arm) and calibration (see the section Needed Effort Using the Sensors), most of the participants would have liked to continue using the CGM, as they got used to it.

#### Numerical Affirmation for Assumed Cause and Effect

Half of the participants (5/10) discussed that they found numerical evidence for the assumptions they had before the study.

These sensors have confirmed my assumptions what are the most important factors to control blood glucose and GDM and weight management in the future...so the regular eating is of paramount importance for me.

Participant #10

Moreover, this included more specific causalities that had been assumed before using the sensors, as Participant 9 found evidence for an association between physical activity and blood glucose.

If you move or plan to move, then you can eat food which has more carbs...so I have been following if I do something I can eat a little bit more...this kind of normal thing that I kind of had thought before...but now it was more like you can actually see it.

Participant #9

### Factors Inhibiting Self-Discovery (RQ1)

Most of the participants (7/10) did not discuss finding cause and effect between physical activity and glucose levels. For example, Participant 9, who was data-oriented, tried to figure out the causalities.

Well, maybe the information from the activity bracelet was useful, as I have never used such a device before and I am interested in numbers...and this information connected to what is happening in my blood glucose...so I tried to figure out connections.

Participant #9

As the self-discovery process seemed to be tedious for many of the participants, they would have liked to receive clear instructions on how to change their behaviors. Some participants wished to see important data being highlighted.

I wished I could have seen highlighting or other markings, what to look for from the data.

Participant #10

As such, the current tools did not support establishing links between glucose levels and physical activity. In the following text, we discuss issues that inhibited self-discovery.

#### Lack of Trackable Features

**Figure 2:**
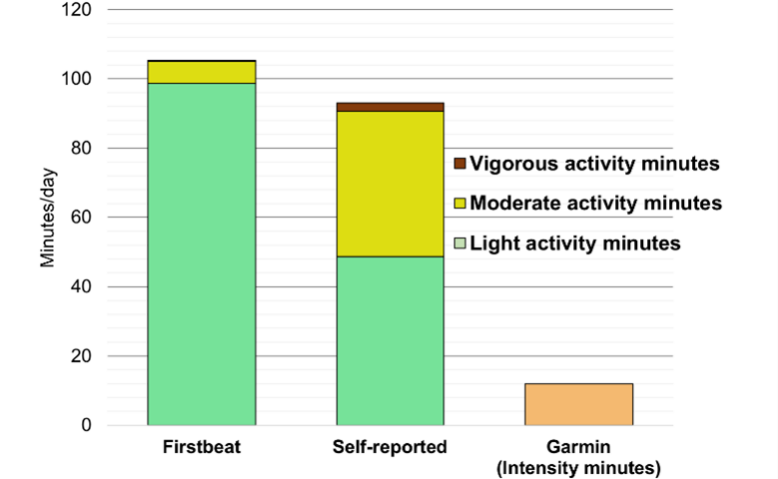
Duration and intensity of daily physical activity as measured with Firstbeat (heart rate variability) and as self-reported. A substantial portion of physical activity that heart rate variability measured to be light was perceived as moderate.

Participants had less physical activity than recommended during the measurement period, as measured with Firstbeat. According to the recommendation, pregnant women should have at least 150 minutes of moderate physical activity in a week [79], but according to Firstbeat, the participants had approximately 7 minutes per day of moderate physical activity (Figure 2). In most cases, the lack of physical activity was explained by being in the third trimester of pregnancy.

Unfortunately, I did not have much physical activity as I get pain from normal walking...I was tempted to do more, but my condition did not allow it.

Participant #2

Thus, without enough physical activity, it was difficult to interpret the effect on glucose.

As the intensity levels of physical activity were difficult to quantify and recognize, the participants had only very little understanding of what the physical activity shown as intensity minutes meant.

They were very confusing, I did not follow them actively, one day I just realized that I have got more of them, but I did not have any clue what they are based on. On one day I became unwell in a shop, and I noticed that I had received intensity minutes because my heart rate had increased...but it was not something nice.

Participant #4

The number of intensity minutes varied a lot between participants, as 1 participant did not gain intensity minutes at all during the measurement period and 1 participant gained 145 minutes (the goal being 150 minutes per week). Moreover, Vivosmart 3 required physical activity to last 10 consecutive minutes to be counted, which was not often the case for participants as their physical activities were performed for shorter periods, such as walking the stairs.

While the intensity of physical activity was difficult to recognize and the intensity minutes were not achieved much or understood well, steps were easily understood, and step goals provided by sensor applications were achieved more often. However, half of the participants (5/10) did not care about the goal, as walking was perceived to be tedious.

I did not care about the step goals, before pregnancy I could have challenged myself, but now I go for a walk which feels good and that’s it.

Participant #5

Three of the 10 participants discussed the importance of the possibility of tracking swimming and water running, as these were the only exercises they were able to perform well.

For a gestational diabetes patient, swimming is almost the only sport that you can pretty normally do, so the sensor should definitely be one that encourages you to move, especially to swim.

Participant #1

This highlights the importance of the waterproofness of physical activity trackers and the possibility of tracking swimming for women with GDM.

#### Difficulty of the Quantification of Self-Tracking Data

We expected the quantified information through wearable sensors to help in forming hypotheses, as an abstraction to quantifiable units (eg, from a fast walk to the heartbeat) is often required at the hypothesis formulation stage [24]. However, the discrepancy between perceived and measured quantification and clearly erroneous quantification with wearable sensors imposed significant challenges for hypothesis formulation. This study showed a significant difference between measured and perceived quantification of physical activity. Participants interpreted the intensity of physical activity as higher than it was measured, that is, participants perceived light activity as moderate activity. This can be seen in Figure 2, which shows a high proportion of physical activity being light, as measured with Firstbeat. The participants self-reported their overall duration of physical activity rather similarly to Firstbeat. In fact, there was a statistically significant correlation between Firstbeat and self-reports (Spearman *r*_59_=0.43; *P*<.001) in terms of the duration of physical activity. However, the participants categorized the intensity (intensity was instructed according to [69]) of physical activity differently than Firstbeat. There were no statistically significant correlations between self-reported values and the values from Firstbeat when looking at each intensity within the categories.

In general, the participants had difficulties in interpreting what is counted as physical activity.

At this point of pregnancy you move a little, and tasks like fetching the mail is already pretty tough...so it is a bit difficult to say what is counted as exercising and what is not.

Participant #4

As such, perceiving physical activity as more intense than measured might lead to incorrect conclusions about its effect on glucose levels.

#### Contradicting Self-Tracking Data

Differences in the data provided by the sensors caused significant challenges for self-discovery. Regarding physical activity, there were statistically significant differences in the number of steps between the devices, as evaluated with the Friedman test (*χ*^2^_2_=16.22; *P*=.008). These differences were not explained by the differences in how long the sensors were worn, as Vivosmart 3 and Firstbeat were worn for similar durations, but Vivosmart 3 (mean 7191 steps/day) provided twice as many steps as Firstbeat (mean 3519 steps/day). Exsed was in the middle with a mean of 6307 steps/day. Firstbeat required a longer continued movement to start the counter, whereas Vivosmart 3 started counting the steps immediately. It is probably a more desirable strategy to also count the steps during small transitions (eg, in the home), as there were only a few pregnant women who exercised. However, Vivosmart 3 counted movement as steps, even though the participants had not walked.

When I woke in the morning, I had several hundred steps, although I had not walked that much during the night.

Participant #1

Contradictory data between sensors were not only limited to steps, as there was no significant correlation in the duration of moderate physical activity between Firstbeat and the amount of intensity minutes in Vivosmart 3 (Spearman *r*_57_=0.22; *P*=.12).

Regarding sleep, there was a statistically significant difference in the length of sleep between the devices, as evaluated with the Friedman test (*χ*^2^_2_=17.27; *P*<.001). Exsed showed significantly less sleep (mean 7.2 h/night) compared with Vivosmart 3 (mean 7.8 h/night) and Firstbeat (mean 8.0 h/night). These differences raised a lot of questions among participants and decreased the credibility of the data. These responses on contradictory data also reflect the UTAUT responses on incompatibility (see the section General Acceptance Based on the UTAUT). Three participants found that the data provided by the sensors they normally use (activity bracelets by Fitbit, Polar, and Suunto) varied significantly in terms of physical activity and sleep.

In addition, 6 of the 10 participants discussed differences between continuous glucose measurements taken from tissue and fingerstick measurements taken from the blood. The reported differences varied a lot. Some participants reported that the differences were significant.

...a couple of times it [Medtronic] showed that the glucose was low, but it wasn’t that low...at one time it [Medtronic] showed 2.8 [mmol/l], but it was 5.3 [mmol/l, as measured from fingertip].

Participant #4

On the other hand, some reported that the differences were minor.

I don’t think they differed much...looking at the graph you were able to see an increase after eating and during night time it was low, so they seemed to be pretty accurate.

Participant #6

Nevertheless, the differences decreased the credibility of glucose monitoring data.

...the values were somewhat different than taken from fingertip...so it made me think how much I can trust this data.

Participant #9

However, the use of multiple sensors supported gathering a lot of data from many perspectives, with the potential to increase understanding.

#### Challenges in Self-Tracking of Sleep

As pregnancy decreases the quality and length of sleep [80], sleep information could be valuable for women with GDM, as they learn to understand their sleeping disorders. Five of the 10 participants mentioned information about sleeping to be particularly interesting.

On Thursday night I slept two hours and six minutes, so it was pretty interesting to get that kind of readings, but I think it is positive in the sense that it proves that I am not becoming crazy but instead slept too little.

Participant #10

Moreover, these participants discussed that they were interested in the quality of sleep.

It was interesting to look at the sleep graph...so in the early night I had slept deeper and lighter towards the morning, and how you have woken or not woken up.

Participant #9

However, 2 of the 10 participants did not want to get feedback about their sleeping as they knew they had slept too little.

I have not had any possibilities to influence my sleeping during the past month, so it could be a bit depressing information that you have slept lousy...Well, I know that already.

Participant #1

Thus, seeing sleep data was clearly a matter of personal preference.

Participants sometimes had difficulties in estimating at what time they had fallen asleep; thus, objectively measured sleep has the potential to provide unbiased information for the self-discovery process. In general, participants’ self-reported duration of sleep (mean 7.8 h/night) correlated with the duration of sleep measured with the wearable sensors Firstbeat (Pearson *r*_42_=0.58; *P*<.001), Vivosmart 3 (Pearson *r*_42_=0.57; *P*<.001), and Exsed (Pearson *r*_36_=0.55; *P*=.001). Moreover, sleep data gathered through sensors were more comprehensive as participants sometimes forgot to mark the waking and sleeping times in the logbook.

Nevertheless, participants were not able to link their sleep with glucose values, although they tried to increase their understanding of how to manage glucose values.

I am most interested in the quality of sleep and stress levels. And how and if they impact the glucose somehow...my fasting glucose values don’t seem to be within the limits no matter what, so it is the same whatever I eat, so I feel that they are always high.

Participant #8

#### Challenges in Self-Tracking of Stress and Recovery

In general, all participants were curious about their stress levels and how these levels were linked to glucose levels. However, most of them (7/10) had difficulties interpreting the stress data provided by Vivosmart 3. Pregnancy increases the resting heart rate and decreases the HRV [81], which has been used as a measurement for stress [82]. The decrease in HRV due to pregnancy most likely caused Vivosmart 3 to interpret standing as stress, although participants did not feel stressed.

The stress data was confusing. I did not understand how it figured out that I had been very stressed that day. I stood a lot at my workstation, so I wondered if it is so silly that it thinks that I am terribly stressed if I stand.

Participant #3

However, 3 of the 10 participants valued the stress data from Vivosmart 3 as it helped them know whether they recovered from stress.

There was one day when I was using a computer and I had meetings for the whole day, it was very stressful for the body, even though I did not do anything physically...these stress sensors sort of gave me information on what is enough rest for recovery, this was new to me.

Participant #9

Seeing themselves being described as stressed did not seem to make them more stressed but sometimes helped to distinguish between stress and rest.

I was able to look at the stress level, so it concretized when I am like resting and when the stress is high.

Participant #7

Participant 8 discussed that the stress reading from the sensor could be used as an objective value like body temperature, which would make a partner understand the condition.

...at home I can show, look how stressed I am...so you should take care of the child while I’m resting.

Participant #8

Thus, stress data were valued by other means than supporting the self-discovery of glucose levels.

#### Toward Better Tools for Supporting Self-Discovery

Although the participants had received their GDM diagnosis some weeks prior to the study’s measurement period, they were still in the discovery phase [38], meaning that they were figuring out the factors affecting their glucose levels. We found many instances that followed the chosen self-discovery framework [24]. Over half of the participants (7/10) found causalities between nutrition and glucose values in continuous glucose monitoring, and 3 of the 10 participants found causalities between physical activity and glucose values in continuous glucose monitoring. However, these causalities were based on gained experience (ie, the food that was just eaten or the walk that was just taken) and CGM data, but not on the data from lifestyle sensors. This indicates that establishing causalities based on self-tracking data through wearable sensors appears to be too challenging, and better tools (or more support from health care professionals) for interpreting the self-tracking data through wearable sensors are needed. In this study, 6 of the 10 participants commented that they would have liked to have added information in a single app, which would have decreased the amount of redundant data shown.

So that the same information would not be entered in many places, but also the same or overlapping information would not be presented to the user, so you should have one app.

Participant #10

This was also reflected by Participant 9.

So that all the information is visible in one place, and there won’t be many links and sources. So, the challenging thing was what I should write on the paper, what I see on the bracelet...so there should be one place and one way to show this information.

Participant #9

The other issue was that participants had to enter the blood glucose values taken from their fingertips into the Medtronic app, as well as write them down with a pen and paper and report these values to a health care professional. This requires double marking of blood glucose values, which can decrease the motivation to track glucose values in the GDM application in the long term [42]. As such, participants indicated that they wished to have a single application where all the data from lifestyle sensors and the CGM are gathered. This would decrease the amount of redundant and contradictory data, as participants were confused by the differences in the data provided by multiple sensors.

### Experiences of Wearing the Sensors (RQ2)

#### Wearing the CGM on the Arm

Most of the participants (8/10) preferred wearing the CGM on the arm instead of near the navel. One reason was that participants did not like to attach the sensor near the baby.

Now when you feel with your hands your baby moving, it would feel somehow weird if there was something in that place during pregnancy.

Participant #6

Other reasons were that the abdomen was sore and the sensor would be visible to self and others. However, wearing the CGM on the arm caused problems with glucose measurement during the night, as the participants slept on the glucose sensor, which caused the sensor readings to drop below the alarm limit, and this woke up most of the participants (8/10). The participants had to turn off the iPod to silence the alarm, which caused some of the glucose measurements to be missing from the sensor. Thus, the participants could not sleep on the side where the sensor was placed. We tried to avoid this by asking on which side the participant typically sleeps and attaching the sensor on the other side, but this did not always help as some participants slept on both sides.

At this stage of pregnancy...you must sleep on both sides, they are the only poses in which you can sleep, so the position has to be something else than that [the arm]...

Participant #1

While most participants (9/10) preferred not to wear the glucose sensor near the navel, Participant 10 preferred that option. However, this participant hit the glucose sensor at various places, such as a car seat.

For example, I hit it [glucose sensor] on the car seat every time I got in the car or got out of the car it hurt...so I wonder if there is a better place for it.

Participant #10

In fact, 3 of the 10 participants reported the issue of hitting the sensor on various objects, causing some pain in the arm. As such, there was no optimal place where this CGM could be placed. An issue with the stickers holding the CGM is that they can be loosened when swimming, which was an important hobby for 3 participants. In fact, the stickers holding the CGM were loosened in 1 participant, and the sensor got detached when swimming. Therefore, stickers as a fastening mechanism for sensors should be avoided in the long run.

...six days is pretty heavy, so you do not want to take them all with you, so I think, especially when there are these glues, so I would not like to wear them for very long.

Participant #1

#### Wearing the Lifestyle Sensors

Overall, the participants wore the sensors over 80% of the time (ie, over 19 h/day), as shown in Table 1. Participants wore the sensors, except when they were showering or swimming. Sometimes they forgot to wear the sensors, and this was especially the case with Exsed (hip worn), which required a change of position before and after sleeping. In fact, there was a statistically significant difference in measurement durations, as evaluated with the Friedman test (*χ*^2^_3_=8.124; *P*=.04). A post-hoc test using Bonferroni correction [83] revealed that data from Exsed were acquired for a significantly (*P*=.03) shorter time (mean 83% of the time) than from the Vivosmart 3 activity bracelet (mean 93% of the time). No other differences were found between the sensors in terms of measurement durations. The Exsed hip sensor operated with batteries during the whole period and did not require charging; however, the Vivosmart 3 sensor was worn more. Participants had only limited wearing issues with Vivosmart 3. Two participants discussed that it caused some swelling, but no other issues were raised. This was different from Exsed worn on the hip. Information regarding the preference of Exsed was obtained from 9 participants. Of the 9 participants, 4 preferred to wear Exsed on a clip, 2 preferred to wear it with a belt, and 3 did not have a preference. The primary reason participants preferred wearing Exsed on a clip was that it was difficult to adjust the tightness of the belt. When the belt was loose, it easily moved around.

It rolled all the time and fell down, so it was a bit irritating.

Participant #10

Moreover, if it was tight, it pressed uncomfortably.

The belt pressed even more [than the clip], I do not know how much it could have been looser.

Participant #8

The belt was used if no place was available for the clip.

I am wearing a skirt or dress, so the belt has been more natural.

Participant #5

Participants also had issues with the clip, as it chafed the skin.

As I have this belly, it [Exsed] is irritating on the waist. ...I had to fix its [Exsed] position and move it so if I am sitting it is under pressure.

Participant #2

In fact, 5 of the 10 participants reported that they had some issues with wearing Exsed with either the clip or belt. As such, pregnancy decreased the feasibility of using a hip sensor for tracking physical activity. However, the hip sensor was perceived as unnoticeable by some participants as it did not have a user interface and it was worn in the trousers.

You did not notice it at all, so sometimes I forgot that I needed to put it on when I took my trousers off.

Participant #4

#### Needed Effort Using the Sensors

The requirement to calibrate the CGM twice a day was found to be tedious.

It [calibration] was needed surprisingly often...although it did not bother me during the week, but in the long term it could become an issue, all those calibrations if you are somewhere [else than home]...

Participant #2

This influenced the sleep of Participant 4, as this participant needed to wake up in the mornings to calibrate the sensor.

On some mornings, it was irritating that it notified me half an hour before calibration, I thought I could have slept half an hour more.

Participant #4

The other issues that needed substantial effort from participants were keeping the nutrition diary and filling the physical activity logbooks. These would not be feasible in the long term.

Writing the diaries took a lot of time. I could not manage that every day.

Participant #3

These responses support the findings from [42] that the requirement of manually entering physical activity reduces the amount of data significantly in the long run among women with GDM. Even manual start/stop for recording exercises was not used much, as it was easily forgotten.

...it was very difficult to remember to mark the activities, like starting the activity and stopping the activity.

Participant #10

#### General Acceptance Based on the UTAUT Questionnaire

Responses to the UTAUT questionnaire (see results in Multimedia Appendix 1) showed good acceptance of sensors before and after usage. For example, participants agreed with the statement “I would find using the sensors as a good idea” (before: mean 6.0, after: mean 6.1; out of 7, where 7 is “strongly agree”). Participants felt that wearable sensors supported behavior change, and they agreed with the statement “Using the sensors will improve my possibility to make a concrete improvement in my lifestyle” (before: mean 6.0, after: mean 5.9; out of 7, where 7 is “strongly agree”). Participants mentioned that being able to see trends could guide their behavior related to diet and physical activity.

Acceptance was not affected by the usage of the sensors, as there was no statistically significant difference in acceptance before and after usage (evaluated with the Wilcox signed-rank test). The largest difference between before and after usage was in the statement “The sensors are not compatible with the other sensors I use for self-tracking.” Before usage, the study participants disagreed with the statement (mean 2.5), but after usage, they slightly agreed (mean 4.5). Only the participants who were using other self-tracking sensors responded to this statement, so the sample size was too small to conduct a meaningful statistical test. However, the responses in the interviews reflected the change in responses on incompatibility.

I found differences in both activity sensors [Exsed and Vivosmart 3] compared to this my own Polar, which was on my other hand. I changed its settings to correspond with the right arm...it [Polar] gave different readings on activity and steps, although the length of a step was set to the same. It was so mysterious why they differed so much.

Participant #10

Despite this incompatibility with the participants’ existing self-tracking devices, the use of wearable lifestyle sensors together with the CGM was acceptable.

## Discussion

### Principal Findings

This is the first study that aimed to investigate how to support self-management of GDM with wearable sensors in addition to CGMs. Regarding self-discovery (RQ1), we found that the CGM supported the learning of the associations between blood glucose and nutrition, but the wearable sensors measuring physical activity, sleep, and stress did not provide significant support for the learning. The challenges included the dispersion of data among multiple apps, missing trackable features, such as type and intensity of physical activity, and a lack of GDM-specific goals for behavior. From the user experience perspective (RQ2), this study highlighted that the benefits overcame the discomfort and effort when wearing the sensors. There were differences in sensor preference, and a wrist-worn sensor was preferred over a hip-worn sensor and was worn for longer. In general, this study further emphasizes the findings [22,43] that self-tracking among women with GDM should be highly automatic. We discuss these results in the following sections with respect to each RQ.

### Supporting Self-Discovery With Wearable Sensors (RQ1)

#### Feature Selection

Starting from *feature selection* (ie, identification of activities that have an impact on blood glucose), this study highlighted the need to tailor the available features and their presentation with respect to GDM. Women with GDM had difficulties in interpreting and accessing the physical activity features. The activity bracelet required users to perform physical activity at a moderate level for 10 consecutive minutes to be able to see the duration of physical activity, which was not often the case for the women with GDM as they performed small activities, such as short walks. In fact, 2 participants did not achieve intensity minutes at all. This might mean showing light physical activity, for example, in terms of steps. However, there are no official health recommendations for steps among pregnant women, and thus, showing the duration of moderate or vigorous physical activity with respect to health recommendations (150 min/week of physical activity at a moderate level [79]) would be a feasible feature on a weekly basis.

Although we used multiple distinct types of wearable sensors for measuring physical activity, there was a lack of automatic recognition of physical activities (ie, swimming and water running) that are important for women with GDM. This challenge will decrease in the future as the automatic recognition of diverse types of physical activities is improving. However, this challenge of automatic recognition of features related to nutrition will remain for a long time. To cover a wide variety of features, MacLeod et al [27] suggested the use of manual tracking as an aid to automatic tracking. This approach allows tracking a large number of features. However, qualitative studies emphasize that pregnant women are typically overwhelmed [54,84] and that women with GDM face considerable time pressures [84]. As such, we argue that automatic self-tracking is especially important for these user groups. In this study, most efforts were required to keep a food diary with a pen and paper, and less demanding methods were requested. Chung et al [85] proposed a lightweight photo-based food diary to support the collection of nutrition data for clinical visits of patients with irritable bowel syndrome. This photo-based diary approach appears to be promising for women with GDM as well. Peyton et al [54] suggested that self-monitoring of pregnant women can be supported and encouraged, in addition to photographic journals, by using simple designs, such as reminders, and by keeping the techniques for user data input simple. Data collection techniques that are undemanding (eg, checkboxes instead of long text) support a quantifiable format, which is needed in the *hypothesis formulation* process [24].

#### Hypothesis Formulation

With respect to *hypothesis formulation* (ie, formulation of suspected associations with activities and blood glucose), participants experienced difficulties in the quantification of the self-tracking data on physical activity, sleep, and stress. Still, most of the participants were interested in monitoring stress, which plays a significant role in the lives of women with GDM [13,86], and sleep, which allows following sleeping disorders due to pregnancy. Thus, this quantified information about stress and sleep provided value to the participants in terms of providing information about their condition, being part of *documentary tracking* [49]. As such, participants were interested in monitoring their sleep and stress rather than changing them. This was opposite to nutrition and physical activity, which were more related to *goal-driven tracking* [49], and their features (although not based on self-tracking data) were an integral part of the self-discovery process.

The results of this study indicate that quantification by sensors needs to match with quantification by the user so that meaningful hypotheses can be formulated. For physical activity, misperception of intensity is problematic as the rate of change of glucose levels depends on the intensity of physical activity [87], and perceiving physical activity differently may lead to wrong conclusions about its effect. This finding of a discrepancy in the perceived and measured intensity of physical activity is in line with the finding in a previous report [88], where women with GDM estimated the amount of vigorous physical activity to be higher than that measured with a hip-worn accelerometer. These results are opposite to the results of a previous study [32], where users with type 2 diabetes reported a high correlation between self-reported physical activity and the duration of vigorous activity measured with an activity bracelet. This indicates that the discrepancy between perceived and measured physical activity is more prominent among pregnant women than among people with type 2 diabetes. This would mean that the intensity levels should be more clearly defined for women with GDM, and providing feedback during the activity (eg, “Now you are swimming at the moderate level.”) would be a good approach. Moreover, the quantification of features with wearable sensors was unreliable, for example, participants could not rely on stress data, which were affected by decreased HRV due to pregnancy. Thus, we agree that more advanced techniques are required to differentiate between the decreased HRV caused by pregnancy and decreased HRV due to stress [59].

#### Hypothesis Evaluation

For *hypothesis evaluation* (ie, evaluation of how the latest information about associations fits with existing knowledge), we observed the challenges of scattered and conflicting data. At this stage, we expected that having the wearable sensors would have facilitated hypothesis evaluation, as there is more data available and its quantified form enables quantitative comparison against existing data. However, we found 2 major reasons why this stage was difficult for the participants. First, the data were scattered across different apps, making comparisons between lifestyle and glucose tedious. The dispersion of data has been identified as a challenge in personal informatics [26,89,90], and this study further emphasized that there should be integrative tools to support self-discovery. Second, the data were contradictory between sensors in multiple ways. For example, there was a statistically significant difference in the number of steps and the duration of sleep between sensors. Moreover, the discrepancies between CGM and fingerstick measurements caused confusion regarding how much participants could rely on CGM data. The discrepancies in data were not limited to the given sensors but extended to participants’ existing sensors (see the section General Acceptance Based on the UTAUT). These discrepancies directed the attention of women with GDM from self-discovery to evaluation of these differences. While the use of multiple sensors potentially increases the reliability of the data, the use of a single sensor for each lifestyle variable would be more appropriate to support reflection. Then, the attention of the user would not be on looking at differences in the data between sensors, but rather on evaluating the impact of activities on glucose levels between instances, such as small variations in meals and physical activities. Moreover, the relative differences in data within a single wearable sensor would provide useful information. However, we acknowledge that trackable features may be unknown for people with chronic illnesses, especially in *poorly understood conditions* [27]. Thus, figuring out the relevant features may require the use of multiple wearable sensors to gather various aspects of chronic illnesses. However, in that case, the data from multiple sensors should not be conflicting but rather supportive for increasing the understanding of the chronic condition.

#### Goal Setting

The goal for women with GDM is simple. The fasting glucose value in the morning should be less than 5.5 mmol/L, and the glucose value 1 hour after a meal should be under 7.8 mmol/L. However, this is a very high-level goal, which participants try to transform into concrete behavioral goals. For the *goal specification* (ie, identification of future goals based on activities and outcomes) phase in self-discovery, we found that participants primarily created goals based on continuous glucose monitoring and experience. Of all the target behaviors, changing the diet was the one that the participants seemed to be the most optimistic about, and they could name several ways of changing it. For example, Participant 1 defined a goal of eating nuts in a meal as the participant figured out that this helps to keep the glucose level below the maximum limit. To help in goal setting, this participant with GDM should know how many nuts or how many grams of nuts to include in the meals and should have a tool to track this goal, which should be developed following a goal-directed self-tracking approach [91]. Transformation of goals defined by the participants (eg, eating more nuts by Participant 1) into features, which are possible to track with wearable sensors, is still a major challenge.

Goals provided by wearable sensors (eg, 150 min/week of physical activity at a moderate or vigorous level) are related to general guidelines and are not specific to the management of GDM. This decreases their value for women with GDM. Some limitations are part of every chronic illness, and individuals with a chronic illness should not be pushed too hard to achieve the goals, as there is a risk of causing *goal frustration* [92], if it is impossible to achieve the goals due to implications from their illness. The goals should be concrete (eg, “walking for 30 min at a moderate level would decrease your glucose levels”) and trackable with wearable sensors. Another type of goal specification we observed was that participants defined goals to collect further evidence for their hypothesis. For example, for Participant 3, the goal was to climb stairs to see whether this had a real impact on glucose levels. Again, this goal should be trackable. Half of the participants (5/10) discussed that they would be willing to change behaviors for physical activity. One reason was that physical activity was measured in a straightforward way (ie, steps) and was experienced as more tangible by the participants than the target behaviors related to sleep and recovery (see the section Challenges in Self-Tracking of Sleep and the section Challenges in Self-Tracking of Stress and Recovery).

One way to approach this would be to provide options for concrete goals, where women with GDM could choose the most preferred goals. Having such a set of options for goals would ease the tracking with wearable sensors, as the number of trackable features and goals could be narrowed down to certain options. Harrison et al [93] suggested having practical options for goals for encouraging physical activity among women with GDM, as the authors found that women with GDM wish to have clear goals for physical activity while still retaining autonomy. We made similar observations for nutrition goals. The requested goals did not only include what to eat considering the diet limitations (eg, due to pregnancy) but also when to eat. This reflects the wish of people with type 2 diabetes, who have experience with continuous glucose monitoring, to have more knowledge on the effect of meals on temporal glucose patterns [94]. While we made the same observation, the women with GDM wished to have concrete suggestions on how to influence these glucose patterns by the content and timing of the meals.

### Experiences of Self-Tracking With Wearable Sensors (RQ2)

We learned that the body placement of sensors is a key factor in acceptability, quality of measurements, and preference, and ultimately a challenge for collecting data. Wearing the physical activity sensor on the wrist, instead of on the hip, has several benefits for pregnant women. Half of the participants (5/10) had issues with wearing the sensor on the hip, as it moved around or chafed the skin when sitting. The drawback of a wrist-worn sensor is that it is not possible to recognize whether the user is sitting or standing. A sensor worn on the hip can recognize this [63]. However, the regulation of sitting and standing relates more to long-term health and health risks [95] rather than to the management of GDM. The sensor on the wrist was worn significantly more than the sensor on the hip, providing more data to the user. Although the hip-worn sensor was used less than the activity bracelet, it was still worn for more than 10 hours a day, which is the minimal duration to obtain credible data [96].

Wrist-worn sensors are particularly feasible for pregnant women, as bracelets can be adjusted with respect to swelling. This is not the case with activity rings, such as Oura, which are not easily worn during pregnancy owing to swelling of the fingers [97]. While there are wearability issues with wrist-worn devices among pregnant women, such as smartwatches if they are heavy [97], the activity bracelet used in the study did not raise issues beyond slight irritation of the skin. This finding has evidence from a long-term study conducted with a similar activity bracelet among pregnant women [98].

The lifestyle sensors were highly accepted among women with GDM. This result extends the finding by Scott et al [46] that CGMs are highly accepted in self-tracking during pregnancy. Women with GDM seem to be less concerned about using wearable sensors compared with people having chronic illnesses, such as chronic heart patients who have had feelings of uncertainty, fear, and anxiety [99]. In our study involving women with GDM, the clear purpose of the wearable sensors (supporting self-discovery and healthy behavior) could have increased the acceptability of the sensors. This was the opposite in the case of heart patients, where the purpose of the sensors was to gather “self-tracking of activity data in relation to their embodied condition and daily practices of dealing with a chronic heart condition” [99]. Thus, clear framing of the purpose of wearable sensors and supporting the goals of the user (in this case, management of glucose levels) with wearable sensors seem to increase the acceptability of self-tracking.

Although the data provided by the CGM was highly valued among participants, most of the participants (8/10) had issues wearing the CGM. Most of the participants (9/10) preferred wearing the CGM on the arm, instead of having it near the navel, which is the primary placement location for the sensor. Wearing the sensor on the arm caused false alarms of glucose levels dropping too low because women with GDM slept on top of the sensor. As such, if the CGM is worn on the arm, a more robust sensor that can overcome pressure issues is needed as pregnant women tend to sleep on their side, at least when over 30 weeks into gestation [100]. Moreover, due to placement on the arm, the participants could not attach the sensor themselves. This decreases the feasibility of using this CGM in the long term, as the CGM needs to be recharged once a week and the sensor can detach, for example, due to swimming (see the section Wearing the CGM on the Arm).

To support self-management, having a single “output” (ie, a GDM application where all the collected data would be shown in a single view; see the section Toward Better Tools for Supporting Self-discovery) also induces the question of having a single “input” (ie, a wearable sensor that collects all the data). A feasible approach would be adding lifestyle tracking capabilities to continuous glucose monitoring. This kind of sensor does not exist yet. An integrated sensor would decrease the problems of wearing and managing multiple sensors, and the data would be recorded in synchrony and without discrepancies, thus helping in establishing the causalities between lifestyle and glucose levels. Moreover, having a single sensor would remove the technical work required to integrate data from multiple cloud services [101]. Ultimately, this integrated sensor would be worn on the wrist. Having a wrist-worn sensor would overcome the difficulties associated with wearing the CGM behind the arm (which can cause false alarms during the night) and wearing the physical activity sensor on the hip. However, noninvasive glucose tracking from the wrist shows poor accuracy resulting from movement, exercise, and sweating [102]. Thus, an optimal solution for a single wearable sensor is yet to be developed.

While we focused on self-discovery without the help of health care professionals, they were very often mentioned. The continuous data collected by the wearable sensors provide an opportunity for remote monitoring and feedback by health care professionals [60]. The participants discussed the importance of having contact with a diabetes nurse, so that they can share the data with them and discuss the data provided by the glucose sensor. This is in line with previous findings that people with a chronic illness need help from experts in the self-discovery process [24,27] and in behavior change. This is further supported by reviews on technological support for diabetes management, which emphasize the importance of 2-way communication between people with diabetes and health professionals [103,104]. Further, self-tracking with wearable sensors can increase the completeness of the self-tracking data presented to health care professionals [105] and can increase the perceived usefulness of the sensors [103,104]. Thus, at this stage, having a 2-way channel between women with GDM and diabetes nurses (eg, through a text chat as suggested by 1 participant) would be a crucial factor in supporting the management of GDM.

Although no wearable sensor other than the CGM supported self-discovery, the sensors increased self-awareness of one’s own lifestyle, and women with GDM believed that this would help them to improve their habits. Thus, wearable sensors have the potential to support behavior change for women with GDM, as self-tracking itself has been found to be an effective behavior technique among people with type 2 diabetes [52]. However, participants discussed that behavior change should be facilitated with recommendations, which would be formulated either automatically based on self-tracking data or manually by health care professionals, and further, the use of artificial intelligence approaches can increase the understanding of cause-and-effect relationships [55,106]. This understanding can be used for setting personal goals for lifestyle changes among women with GDM [107], which were highly requested by the participants of this study.

### Study Limitations and Future Research

We acknowledge that the number of participants could have been higher. However, the main approach of this study was qualitative, and we believe that the number of participants was enough as no new codes emerged after 8 interviews, indicating the saturation of data. Moreover, the same number of participants has been used in qualitative studies on experiences of GDM (eg, [14]). Quantitative investigations on the acceptance of self-tracking among women with GDM would require a longer usage period with more participants.

Women with GDM wore multiple wearable sensors at the same time in this study, which might have affected their acceptance. Despite this, responses to the UTAUT questionnaire in this study reflected high acceptance of wearable sensors. The high acceptance could have been influenced by the fact that the participants volunteered for the study, and thus, they showed at least some interest in self-tracking and were not afraid of pricking their skin. In fact, 1 participant did not want to participate as this participant heard that the study involves skin pricking. Therefore, the acceptability could be biased, and this is similar to studies investigating the acceptability of CGMs among women with GDM [44-47].

The self-discovery process of GDM is challenging and demanding, which currently takes a considerable amount of time. Carolan-Olah et al [84] investigated how the teaching of GDM could be improved, particularly among women with multiethnic and low socioeconomic backgrounds. Cultural differences may pose a need for different trackable features for GDM, for example, water activities among women (eg, swimming and water running) are less feasible in some cultures [108].

This study focused on CGMs and wearable physical activity sensors. As nutrition is an important factor in the management of GDM, future work should investigate the use of wearable sensors for nutrition tracking. At the current stage, they are not able to detect the intake of macronutrients (eg, carbohydrates) [70,71,109], and thus, their support for self-discovery is expected to be limited. However, research on wearable and nutrition collection methods is very active and should be considered in the future.

We have designed a mobile app according to the results of this study, and we will conduct a long-term clinical evaluation in a randomized controlled trial to explore the effect of comprehensive self-tracking with a mobile app on glucose levels [110].

### Conclusions

We have provided the results of a user-centered design process of a mobile health intervention for supporting the self-management of GDM. Our holistic approach for supporting the self-management of GDM with mobile technology included investigations of wearable sensors and a mobile app from self-discovery (learning) and user experience perspectives. We showed multiple issues that inhibit self-management, such as inadequate support for self-tracking physical activity, data discrepancy, and challenges wearing the CGM. One major challenge was the scatteredness of self-tracking data. To support learning further, visualization with guidance through tips and recommendations should be designed to increase the ability of women with GDM to manage diabetes in their pregnancy. The design should consider pregnancy-specific wearability challenges and requirements for data gathering and representation proposed in this paper.

## Appendix

Multimedia Appendix 1 Responses to the Unified Theory of Acceptance and Use of Technology (UTAUT) questionnaire.

## Abbreviations

CGM: continuous glucose monitor
GDM: gestational diabetes mellitus
HRV: heart rate variability
UTAUT: Unified Theory of Acceptance and Use of Technology
VO2 max: maximal oxygen consumption
