# Supplementary material for: Supporting the Management of Gestational Diabetes Mellitus With Comprehensive Self-Tracking: Mixed Methods Study of Wearable Sensors
Source: JMIR Diabetes. 2023 Oct 31;8:e43979. doi: 10.2196/43979 (PMC10646680; doi:10.2196/43979)
Supplement: Multimedia Appendix 1 [file diabetes_v8i1e43979_app1.docx]

| **Multimedia Appendix 1.** Responses to the Unified Theory of Acceptance and Use of Technology (UTAUT) questionnaire | | | | |
| --- | --- | --- | --- | --- |
| **Statements measured with a Likert scale**  **(1= strongly disagree, 7 = strongly agree)** | Mean - Before usage | SD - Before usage | Mean - After usage | SD - After usage |
| I would find the sensors useful for supporting healthier lifestyle. | 5,7 | 0,9 | 6,1 | 0,7 |
| Using the sensors enables me to improve my lifestyle more quickly. | 5,4 | 1,2 | 5,8 | 1,2 |
| Using the sensors makes me live healthier. | 5,7 | 0,8 | 5,8 | 0,9 |
| If I use the sensor, I will increase my chances of getting a concrete lifestyle improvement. | 6 | 0,9 | 5,9 | 0,9 |
| My interaction with the sensor would be clear and understandable. | 5,3 | 1,2 | 5,5 | 0,8 |
| It would be easy for me to become skillful at using the sensors. | 5,7 | 1,3 | 5,8 | 1,1 |
| I would find the sensors easy to use. | 5,2 | 1,4 | 5,6 | 1,3 |
| Learning to operate the sensors is easy for me. | 5,3 | 1,7 | 5,6 | 1,1 |
| Using the sensors is a good idea. | 6 | 1,2 | 6,1 | 0,7 |
| The sensors make changing lifestyle more interesting. | 5,8 | 1,6 | 5,9 | 1,1 |
| Using the sensors is fun. | 5,4 | 1,3 | 5,4 | 1,5 |
| I like using the sensors. | 5,4 | 1,3 | 5,5 | 1,5 |
| People who influence my behavior think that I should use the sensors. | 3,7 | 1,3 | 4,2 | 1,8 |
| People who are important to me think that I should use the sensors. | 3,7 | 1,3 | 4,3 | 1,6 |
| The study nurses have been helpful in the use of the sensors. | 6,4 | 0,7 | 6,3 | 0,8 |
| In general, the study nurses have supported the use of the sensors. | 6,5 | 0,7 | 6,2 | 0,6 |
| I have the resources necessary to use the sensors. | 6 | 1,2 | 6,1 | 0,6 |
| I have the knowledge necessary to use the sensors. | 5,8 | 1,1 | 5,9 | 0,9 |
| The sensors are not compatible with my other sensors I use for self-tracking. | 2,4 | 0,5 | 4,5 | 2,8 |
| A specific person (or group) is available for assistance with sensor difficulties. | 6,4 | 0,8 | 6,1 | 1,2 |
| I could improve my lifestyle using the sensors If there was no one around to help me with sensors. | 5,3 | 1,3 | 5,9 | 1,7 |
| I could improve my lifestyle using the sensors If I could call someone for help if I got stuck. | 6,2 | 0,8 | 6,3 | 1,1 |
| I could improve my lifestyle using the sensors If I had a lot of time. | 6 | 0,8 | 6 | 1,3 |
| I could improve my lifestyle using the sensors If I had just the built-in help facility for assistance. | 4,8 | 1,2 | 5 | 1,8 |
| I feel apprehensive about using the system. | 2,3 | 1,6 | 1,3 | 0,5 |
| It scares me to think that I could lose a lot of information using the system by hitting the wrong key. | 2,4 | 1,6 | 1,6 | 1,3 |
| I hesitate to use the sensors for fear of making mistakes I cannot correct. | 2 | 1,3 | 1,2 | 0,6 |
| The sensors are somewhat intimidating to me. | 1,2 | 0,4 | 1,3 | 0,7 |
